# Molecular docking-guided discovery of *Alhagi maurorum* metabolites as dual-target biofungicides against *Cercospora* leaf spot in sugar beet

**DOI:** 10.1007/s12298-026-01728-0

**Published:** 2026-03-24

**Authors:** Reham I. Abdel Hamid, E. M. Naema Salama, Ibrahim S. H. El-Gamal, Samar A. M. Helmy, Mohamed Elshafiey, Ahmed G. Soliman, M. S. Abbas

**Affiliations:** 1 Sugar Crops Research Institute, Agriculture Research Center, Giza, Egypt; 2 Plant Pathology Department, Faculty of Agriculture, Al-Azhar University, Cairo, Egypt; 3 Biotechnology Program, Faculty of Agriculture, Ain Shams University, Cairo, Egypt

**Keywords:** *Cercospora beticola*, *Alhagi maurorum*, Botanical fungicide, Molecular docking, Sustainable agriculture, Integrated disease management, Plant–pathogen interaction

## Abstract

*Cercospora* leaf spot (CLS), caused by *Cercospora beticola*, is a severe threat to sugar beet (*Beta vulgaris*) production in Egypt’s Nile Delta and poses resistance and ecological problems to synthetic fungicides. This study investigates *Alhagi maurorum* (camelthorn), an endemic medicinal shrub of arid regions, and evaluates it as a sustainable plant-sourced biofungicide for integrated CLS management. Following molecular confirmation of *C. beticola* (PCR amplified actin gene, 959 bp; ITS region sequenced and deposited in GenBank under accession PX884367), *A. maurorum* ethanolic extract (GC–MS) profiling of the ethyl iso-allocholate (1.39%), squalene (7.81%), and methyl oleate (27.33%) was determined as the antifungal constituents from GC–MS profiling. Molecular docking highlighted ethyl iso-allocholate’s dual inhibition of CYP51 (− 8.4 kcal/mol) and CHS (− 6.5 kcal/mol), critical for fungal ergosterol biosynthesis and cell wall integrity. In vitro assays demonstrated complete fungal suppression at 10% extract concentration, mirroring the synthetic fungicide Score®. The efficacy of the extract in controlling disease (66.4–71.5% disease control), along with increased root yield (34.23 tons/fed) and sucrose purity (91.81–92.38%) in two-season field trials, was validated in CLS endemic Kafr EL Sheikh. The biochemical analyses of treated plants showed elevated antioxidant defenses, reflected in phenolics (184.43–186.73 mg/g) and superoxide dismutase (SOD: 122.10–123.77 U/g), to protect the plants against oxidative stress. As an eco-friendly alternative to synthetic fungicides, the extract has biodegradability, pharmacokinetic suitability, and multi-target action. By bridging phytochemistry, computational biology, and field agronomy, this work advances integrated, climate-resilient strategies for CLS management and underscores the potential of arid-adapted plants in sustainable agriculture.

**Supplementary Information:**

The online version contains supplementary material available at 10.1007/s12298-026-01728-0.

## Introduction

A significant source of commercially produced sucrose in the world, sugar beet *(Beta vulgaris L*.) supplies approximately 20% of the world’s supply of sugar (Stevanato et al. 2019; Dohm et al. 2014). However, its cultivation is threatened by its high susceptibility to Cercospora leaf spot (CLS), due to the fungal pathogen *Cercospora beticola Sacc*. Under severe epidemics, climatically limited, necrotic lesions on foliage inhibit photosynthesis by more than 50%, reduce root yield and sucrose content by 30–50%, and severely impact photosynthesis and yield (Ata 2007, 2014; Gouda et al. 2022; Tan et al. 2023). CLS severity rates in Egypt’s Nile Delta reach 45–75%, and these areas of Egypt, such as Kafr El-Sheikh and Dakahliya, are additionally affected by warm, humid microclimates that promote pathogen proliferation (Ata 2014).

CLS is widely treated with synthetic fungicides like difenoconazole (Score®), which have been extensively used. However, such continuous applications pose concerns of environmental contamination, residual toxicity, and an increase in fungicide resistance in *C. beticola* populations (Rangel et al. 2020). These issues are coupled with rising production costs (Fatouh et al. 2011; Fisher et al. 2018). Hence, alternative disease management strategies must be developed to meet these challenges.

Plant-derived bioactive compounds offer alternatives for biodegradability, reduced environmental persistence, and different modes of action (Fatouh et al. 2011; Isman 2020). *Alhagi maurorum* (camelthorn) is a perennial shrub endemic to North Africa and the Middle East that has been traditionally used for medicinal purposes and has a rich phytochemical profile with potential antimicrobial properties towards phytopathogens that are yet to be fully explored (AL-Snafi 2016).

Gas Chromatography-Mass Spectrometry (GC–MS) profile aided in determining *A. maurorum’s* bioactive compounds and provided clues to the possible identification of antifungal agents (Adams 2007). This approach is complemented by molecular docking studies that elucidate possible inhibitory mechanisms (in a biological context) against enzymes of importance in fungi. Lanosterol 14α-demethylase (CYP51), a cytochrome P450 enzyme critical for commercial fungicides, because it is essential for ergosterol biosynthesis in fungal cell membranes, and chitin synthase (CHS) is a target for fungicides as they are essential for wall integrity of fungal cells (Loper 1992). Physicochemical properties, ADME, and pharmacokinetic parameters can also be assessed in silico on compound bioavailability, stability, and toxicity without extensive experimental testing (Lipinski et al. 2001; Pires et al. 2015).

To examine *A. maurorum's* antifungal potential against *C. beticola*, the present study used an integrated approach: (1) isolation and molecular confirmation of the pathogen; (2) GC–MS chemical profiling; (3) molecular docking against CYP51 and CHS; (4) in silico ADME predictions; (5) in vitro antifungal assessment by CLSI guidelines (CLSI 2017) followed; (6) greenhouse efficacy evaluation; and (7) field validation trials. We also looked at the effect of the extract on plant biochemical defenses, photosynthetic pigments, and quality parameters of sugar beet.

This work bridges the gap between phytochemistry, computational biology, and agronomy to bridge the technology development of botanical fungicides for integrated disease management (FAO, 2022; Lamichhane et al. 2016). These findings serve to reduce reliance on synthetic fungicides and develop a practice representing a sustainable approach for the protection of sugar beet productivity in CLS endemic areas.

## Materials and methods

### Isolation and identification of the causal organism

Samples of sugar beet leaf with *Cercospora* leaf spot symptoms were collected from Kafr El Sheikh Governorate, Egypt, in clean plastic bags and brought to the laboratory. They were therefore carefully washed with running tap water, air dried, and lesion areas cut into small pieces. The fragments were surface sterilized in 3% sodium hypochlorite solution for 3 min, rinsed thrice with distilled water, and dried on the sterilized filter papers. Tissues were transferred to Petri dishes containing sugar beet leaf extract dextrose agar (SBLDA) media (100 mL sugar beet leaf extract, 20 g sucrose, 15 g agar per liter distilled water). Fungal growth was examined daily by incubating the plates at 27 ± 2 °C.

Microscopically, growing fungi were examined and purified by a single spore technique (Dhingra and Sinclair 1995; Morsy et al. 2022). Fungal isolates were identified based on the morphological characteristics according to (Barnett 1960; Domsch et al. 1980). The Plant Diseases Survey Department, Plant Pathology Research Institute, Agricultural Research Center, Giza, Egypt, confirmed the identification. For further study, the cultures were maintained on PDA slant medium (200 g potato extract, 20 g dextrose, and 15 g agar per liter distilled water) at 5 °C.

### Molecular confirmation of Cercospora beticola

Genomic DNA from fungal mycelia was extracted using the Quick Plant Genomic DNA Extraction Kit (Qiagen) according to the manufacturer’s protocol. Species-specific primers were used in PCR amplification using primers *CBACTIN959L* (*5′-AGC ACA GTA TCA TGA TTG GTA TGG-3′*) and *CBACTIN959R* (*5′-CAC TGA TCC AGA CGG AGT ACT TG3′)* that target a 959 bp fragment of the actin gene (Larteyl et al*.,* 2003). The reaction mixture (25 µL) contained 12.5 µL PCR master mix, 1 µL of each primer (10 µM), 1 µL DNA template, and 9.5 µL nuclease-free water. The thermal cycling conditions were: 95 °C for 2 min, followed by 30 cycles of denaturation (95 °C, 1 min), annealing (60 °C, 1 min), extension (72 °C, 1 min), and final extension (72 °C) for 7 min. The PCR products were then resolved on a 1% agarose gel and stained with Gold View™ and a 50 bp DNA ladder. This was confirmed by a band of 959 bp for *Cercospora beticola.*

To provide unequivocal molecular confirmation, the internal transcribed spacer (ITS) region of the rRNA gene cluster was amplified using universal fungal primers ITS1 (5′-TCCGTAGGTGAACCTGCGG-3′) and ITS4 (5′-TCCTCCGCTTATTGATATGC-3′) (White et al. 1990). The PCR product (~ 650 bp) was purified and sequenced bidirectionally using Sanger sequencing. The obtained sequence was compared with those in the NCBI GenBank database using BLASTn, confirming 99.8% identity with Cercospora beticola (Accession No. PX884367). The sequence has been deposited in GenBank under accession number PX884367.

### Plant material and extract preparation

#### Plant collection and processing

The aerial parts of *Alhagi maurorum* Medik. *var. maurorum* (Family: Fabaceae) were collected from Ismailia Governorate, Egypt, during the flowering stage. Fresh plant material was washed with distilled water, shade-dried for 14 days at 25 °C, and ground in a mechanical grinder.

#### Extract preparation

A Soxhlet apparatus was employed for sequential extraction with ethanol (70% v/v) at 60 °C for 8 h. A crude extract was obtained by evaporating the solvent under reduced pressure using a rotary evaporator (Büchi R-300). For experimental treatments, the extract powder was soaked in sterilized distilled water at concentrations of 2.5%, 5%, 7.5%, and 10% (w/v) for 24 h, then filtered through Whatman No. 1 filter paper and sterilized through a Millipore filter (0.45 µm). The extracts were stored at -20 °C until further analysis.

### GC–MS analysis and compound identification

#### Instrumentation and parameters

Chemical composition analysis was performed by a quadrupole mass spectrometer Trace GC-TSQ (Thermo Scientific, USA), equipped with TG5MS capillary column (30 m × 0.25 mm × 0.25 µm). The oven temperature was started and held at 50 °C (2 min) for 2 min; ramped to 250 °C at 5 °C/min (2 min) to 300 °C at 30 °C/min (2 min hold). 1 mL/min was the flow rate of helium carrier gas. Injector and transfer line temperatures were 270 °C and 260 °C, respectively. 1 µL of the sample was injected in split mode through the AS1300 autosampler, and 4 min was taken as solvent delay. EI mass spectra were obtained (70 eV) over m/z 50–650; compounds were identified using the NIST 14 and WILEY 09 databases.

### Molecular docking studies

#### Target enzymes

Two fungal enzymes critical to *Cercospora beticola* survival were selected: Table 1.

**Table 1:** Target fungal enzyme for antifungal activity studies

Enzyme	Full Name	PDB ID	Function
CYP51	Lanosterol 14α-demethylase	5FSA	Involved in ergosterol biosynthesis
CHS	Chitin synthase	6TWR	Essential for cell wall integrity

*PDB* Protein Data Bank, *CYP51* Cytochrome P450 Family 51, *CHS* Chitin synthase

#### Ligand preparation

The 3D structures of the five prioritized compounds were retrieved from PubChem and ChemSpider (https://www.chemspider.com/), optimized using Open Babel (v3.1.1) (O'Boyle et al. 2011), and energy-minimized with the MMFF94 force field.

#### Docking protocol

Docking was performed using AutoDock Vina (v1.2.0) in PyRx (v0.8) (Kondapuram et al. 2021). Grid boxes centered on active sites were defined as:

- CYP51: X = 63.39, Y = 59.02, Z = 68.40 (size: 25 × 25 × 25 Å).
- CHS: X = 64.74, Y = 54.58, Z = 451.51 (size: 30 × 30 × 30 Å).

Exhaustiveness was set to 8, and the top 10 poses were analyzed. Binding interactions were visualized in Discovery Studio 2022. Additional validation was performed using CB-Dock2, an improved version of the CB-Dock server for protein–ligand blind docking, integrating cavity detection, docking, and homologous template fitting (Yang et al. 2022a, b).

### Physicochemical, ADME, and pharmacokinetic predictions

#### Swiss ADME analysis

The Swiss Institute of Bioinformatics (SIB) offers the free Swiss ADME web tool, which can be employed to assess the physicochemical characteristics, forecast pharmacokinetic features, and anticipate the Absorption, Distribution, Metabolism, and Excretion (ADME) parameters of authenticated compounds. Thus, SMILES notations regarding the proven compounds' chemical structures were imported to the Swiss ADME web tool's online server for further calculation processing (Daina et al. 2017). Besides, the investigated compounds’ toxicity features were examined employing the pharmacokinetics of small molecules (pkCSM) descriptors algorithm tool (Pires et al. 2015).

### In vitro* antifungal assay*

#### Effect of plant extract on linear growth

PDA medium containing different concentrations of *Alhagi* extract (2.5%, 5%, 7.5%, and 10% w/v) was prepared in sterilized conical flasks. For the fungicide treatment, Score® was added at 0.5 mL/L to the PDA medium. Control plates contained PDA medium with no additives. Each treatment was replicated five times. All Petri dishes were inoculated with 5 mm diameter mycelial disks cut from 14-day-old cultures of *C. beticola* and incubated at 27 ± 2 °C for 14 days or until control plates were fully colonized. The diameter of developed colonies was measured in centimeters. Percentage of fungal growth reduction (X) was calculated using the formula suggested by Abd-El-Moity 1985: X = [(G1-G2)/G1] × 100, where G1 is the linear growth of the pathogen in control, and G2 is the linear growth in treated plates.

#### Disc diffusion method

Agar plates (PDA) were inoculated with fungal mycelial discs (5 mm). Sterile filter paper discs impregnated with *Alhagi* extract (10 mg/mL) or pure compounds (dissolved in DMSO) were placed on the agar. Plates were incubated at 27 °C for 7 days. Inhibition zones (mm) were compared to controls (DMSO and Score® fungicide).

#### MIC determination

The broth microdilution method assessed the minimum inhibitory concentration (MIC). Serial dilutions of compounds (0.1–10 mg/mL) were tested in potato dextrose broth (PDB). Fungal growth was measured spectrophotometrically (OD₆₀₀) after 72 h.

### Greenhouse experiment

#### Experimental design

A randomized complete block design (RCBD) with three replicates was used to evaluate the efficacy of *Alhagi* extract against *Cercospora beticola*. Treatments included:

**T1**: Water control **T2-T5**: *Alhagi* extract (2.5%, 5%, 7.5%, 10% w/v).

**T6**: Commercial fungicide (Score®; 0.5 mL/L). Each treatment in one replicate consisted of 5 plants, one per pot.

#### Plant cultivation and inoculation

Sugar beet (*Beta vulgaris* cv. Rass poly) seeds were sown in pots filled with sterilized soil (sand: peat: clay, 1:1:1). Plants were grown under controlled conditions (27 ± 2 °C, 70% humidity). Treatments were foliar-sprayed at 75-, 90-, and 105-days post-planting (DPP). Two days post-spraying, plants were inoculated using a handheld atomizer with *C. beticola* conidial suspension (3 × 10^5^ spores/mL) (Crane and Calpouzos 1984). Inoculated plants were covered with polyethylene bags for 48 h to maintain humidity.

#### Disease assessment

Disease severity was scored 14 days after the final spray using a 0–5 scale (Shane and Teng 1992):

0: No symptoms,5: > 75% leaf area necrotic.

Treatment efficacy (%) was calculated as: Efficacy = [(D control – D treatment)/D control] × 100, where D control = disease severity in untreated control plants and D treatment = disease severity in treated plants (El-Shabrawy & Abd Rabboh 2020).

### Field experiment

#### Experimental setup

Two field experiments were conducted on sugar beet at Sakha Research Station (latitude of 31° 05′ 39″ N and a longitude of 30° 56′ 31″ E, with an elevation of 4.07 m above sea level), Kafr El-Sheikh Governorate, Egypt, in 2022/2023 and 2023/2024 seasons. The experiment followed an RCBD with three replicates. Plots (21 m^2^) consisted of five ridges with a length of 7 m, a width of 60 cm, and a hill spacing of 20 cm, sown with sugar beet cv. Rass poly. Treatments included:

- **T1**: Water control, **T2**: Score® fungicide (0.5 mL/L), **T3**: *Alhagi* extract (10% w/v)

#### Application and disease assessment

Treatments were applied three times (75, 90, 105 DPP). Disease severity was assessed on 50 plants/plot using the scale of Shane and Teng (1992). Treatment efficacy was calculated as described in Sect. 8.3.

#### Growth parameters

Two weeks after the last spraying, ten random plants per plot were sampled to determine photosynthetic pigments (chlorophyll a, b, and carotenoids) according to Wettstein (1957). The root and top fresh weight (g/plant) of ten random plants per plot were measured at harvest.

#### Biochemical assessment

Two weeks after the last treatment application, samples were collected from 10 randomly selected plants per plot. Total phenols (mg/g fresh weight) were determined according to (Bray and Thrope 1954). Superoxide anion content was calculated using Zhang's method 2007. Superoxide Dismutase (SOD) activity was estimated by the method of Beyer and Fridovich (1987). Catalase activity (CAT) was determined using Aebi's method (1984). Enzyme activity levels were evaluated as units per g of protein content (U/g protein).

#### Quality parameters

At harvest, 10 roots per plot were sampled to measure quality traits. Sucrose percentage was estimated in fresh sugar beet root samples using a Saccharimeter according to A.O.A.C. (1995). Purity percentage was calculated according to Devillers (1988). The percentage of sugars lost to molasses (SLM) was determined using the equation: SLM% = [0.14(Na + K) + 0.25(α amino N) + 0.5]. Extracted sugar percentage was calculated as: Extracted sugar% = sucrose%—SLM%—0.6 (Dexter et al. 1967). Reducing sugar was assessed according to the A.O.A.C. (1990) method. Root impurities (α-amino N, Na, and K) were determined following A.O.A.C. (2005).

#### Yields

At harvest, yield components were assessed from the sampled plants per plot. Root yield (ton/fed.), top yield (ton/fed.), and sugar yield (ton/fed.) were measured from field plots with a planting density of approximately 33,000 plants per feddan. Sugar yield (ton/fed.) was calculated as: Root yield (ton/fed.) × extracted sugar%.

### Statistical analysis

All obtained data were statistically analyzed using the MSTAT-C computer software package. Analysis of variance (ANOVA) for the randomized complete block design was conducted according to Gomez and Gomez (1984). The least significant differences (LSD) method was used to test differences between treatment means at the 5% significance level, as described by Snedecor and Cochran (1980).

## Results

The growing threat posed by the *Cercospora beticola* pathogen, which causes *Cercospora* leaf spot of sugar beets (*Beta vulgaris*), requires a robust counter-strategy to identify and control these pathogens. The present study was undertaken to isolate and confirm the pathogen, assess the antifungal activity of *Alhagi maurorum* extract, and validate its potential using molecular, biochemical, and agronomic evaluations. The methodology included pathogen isolation, molecular confirmation, phytochemical profiling, molecular docking, and in vitro and planta experiments. The results presented here begin with the molecular confirmation of *C. beticola* and continue with these subsequent findings.

### Molecular confirmation of Cercospora beticola

**Fig. 1:**
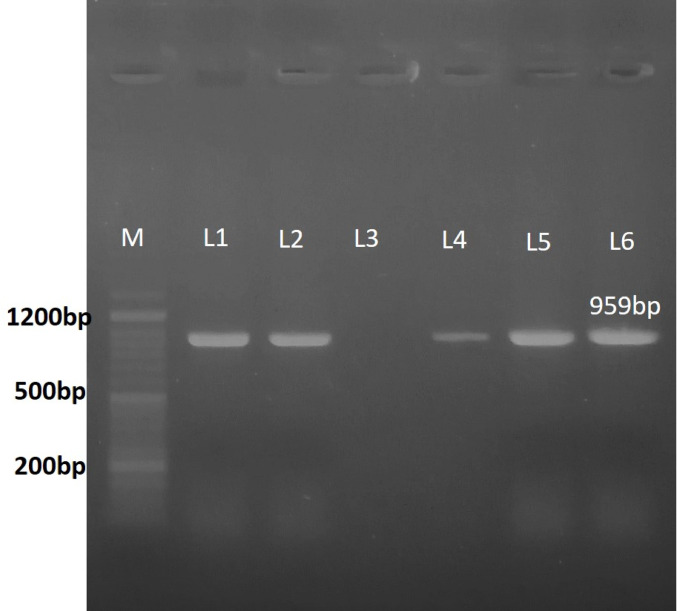
Molecular confirmation of *Cercospora beticola* via PCR amplification of the actin gene. M: 50 bp DNA ladder with labeled bands (200 bp, 500 bp, and 1200 bp). L1, L2, L4, L5, and L6: positive samples showing the specific single 959 bp band corresponding to *C. beticola*. L3: negative sample, showing no amplification. A single distinct band in the positive lanes confirms that the isolates are *C. beticola*, ensuring reliable pathogen identification

The identification of the fungal isolate as *Cercospora beticola* was confirmed by species-specific primers for the actin gene PCR using primers *CBACTIN959L* and *CBACTIN959R*, which yielded a 959 bp band (Fig. 1). These morphological characteristics are consistent with those observed during initial isolation of this ascomycetous species with hyaline, septate hyphae and elongated conidia, which were molecularly validated. For definitive confirmation, the internal transcribed spacer (ITS) region was sequenced, producing a 646 bp sequence that showed 99.8% identity with *C. beticola* reference sequences in the NCBI database. This sequence has been deposited in GenBank under accession number PX884367.

### GC-MS analysis and identification of bioactive compounds in alhagi maurorum extract

GC–MS analysis of *the ethanolic extract of A. maurorum* identified multiple phytochemicals and five essential compounds that inhibit fungal growth (Table 2). Among the other compounds identified, methyl oleate (C₁₉H₃₆O₂), 27.33% of the total extract (RT: 23.20 min; m/z: 296, 264, 222), is the most abundant compound; followed by squalene (C₃₀H₅₀; 7.81%, RT: 32.56 min; m/z: 410, 341), and vaccenic acid (C₁₈H₃₄O₂; 9.12%, RT: 24.67 min; m/z: 282, 264). Minor constituents included 2,3-dihydroxypropyl hexadecanoate (C₁₉H₃₈O₄; 3.46%, RT: 22.20 min; *m/z*: 330, 256) and ethyl iso-allocholate (C₂₆H₄₄O₅; 1.39%, RT: 33.68 min; *m/z*: 436, 382). The mass spectra and retention indices of these compounds were matched against the NIST 17 and Wiley Registry databases. The profile, dominated by lipophilic compounds such as methyl oleate and squalene, suggests the capacity to disrupt fungal membrane integrity.

**Table 2:** GC–MS analysis of extract showing major bioactive compounds with antifungal properties

Compound	RT (min)	Molecular Formula	CAS#	Area %	Key Fragments (m/z)
Methyl Oleate	23.20	C_19_H_36_O_2_	112–62-9	27.33	296, 264, 222, 180, 55
Squalene	32.56	C_30_H_50_	111–02-4	7.81	410, 341, 231, 69, 81
Vaccenic Acid	24.67	C_18_H_34_O_2_	506–17-2	9.12	282, 264, 222, 55, 69
2,3-Dihydroxypropyl Hexadecanoate	22.20	C_19_H_38_O_4_	542–44-9	3.46	330, 256, 239, 171, 129
Ethyl Iso-Allocholate	33.68	C_26_H_44_O_5_	NA	1.39	436, 382, 253, 149, 107

*RT* Retention Time, *CAS#* Chemical Abstracts Service registry number, *m/z* mass-to-charge ratio, *GC–MS* Gas Chromatography-Mass Spectrometry, *NA* Not Available

### Molecular docking and pharmacokinetic profiling of Alhagi maurorum compounds

#### Molecular docking studies

**Fig. 2:**
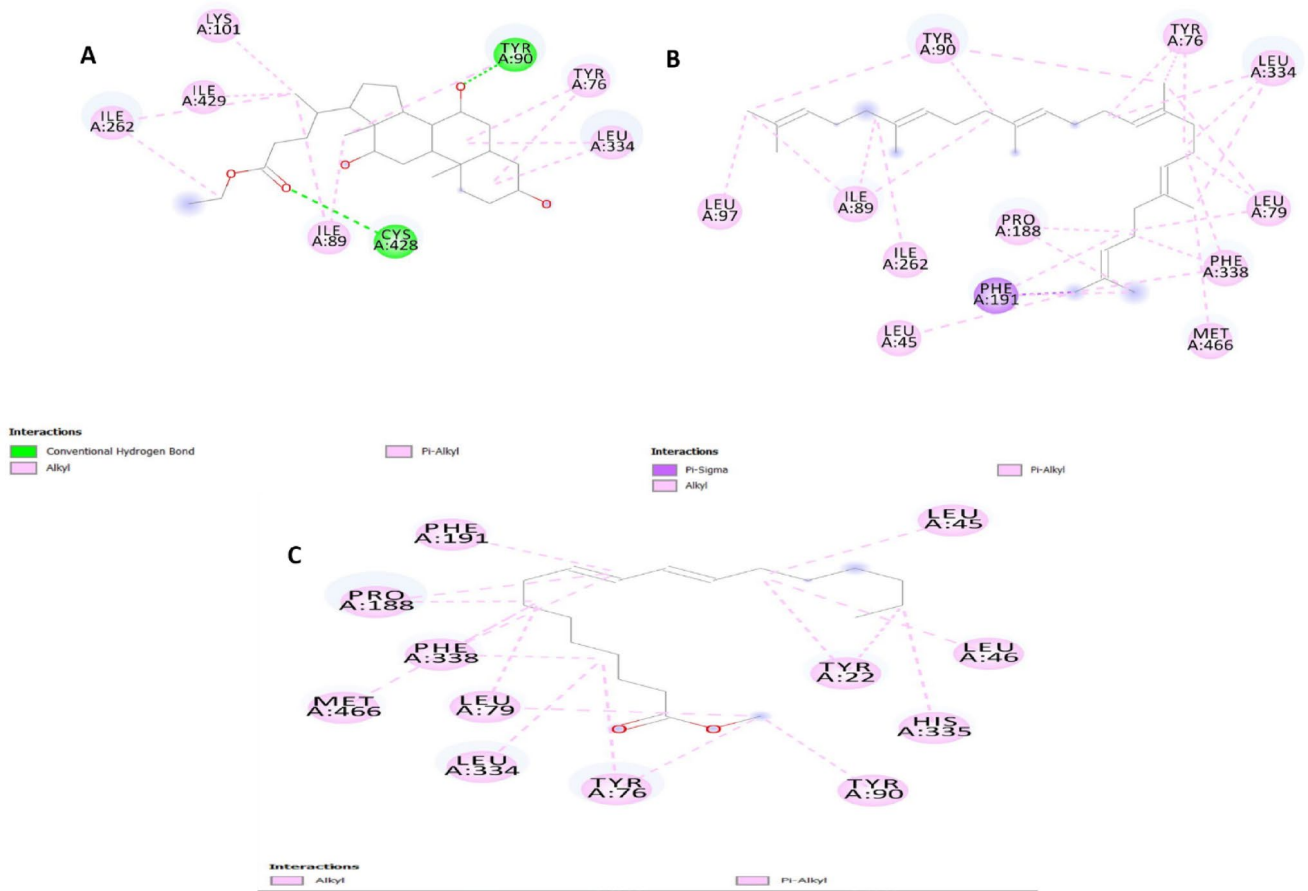
Molecular docking interactions of *Alhagi maurorum* compounds with CYP51 (PDB: 5FSA). **A** Ethyl iso-allocholate (green) forms hydrogen bonds (yellow dashes) with CYS A:428 and TYR A:90, alongside alkyl/π-alkyl interactions (purple dashes) with TYR A:76 and LEU A:334.** B** Squalene (cyan) engages PHE A:191 and MET A:466 via hydrophobic interactions.** C** Methyl oleate (orange) binds to PHE A:338

Molecular docking simulations showed strong interactions between the bioactive compounds of *Alhagi maurorum* and CYP51 (lanosterol 14α-demethylase) and CHS (chitin synthase). As summarized in Table 3, ethyl iso-allocholate exhibited the highest binding affinity CYP51 of the newly synthesized ethyl iso-allocholate was − 8.4 kcal/mol, exceeded the control fungicide difenoconazole − 8.5 kcal/mol, but is almost identical to the control fungicide nikkomycin-Z (− 7.9 kcal/mol) (Fig. 2A). This compound engaged the sterol binding pocket of CYP51 in a way that placed this compound into the pocket in a stereospecific fashion, making conventional hydrogen bonds with CYS A:428 and TYR A:90 and at the same time and in line with π-alkyl interactions with TYR A:76 and LEU A:334 (Fig. 2B, Supplementary File 1). Notably, CYP51 had good affinity (− 7.3 kcal/mol) for squalene, which likely results from π-sigma and hydrophobic interaction with the PHE A:191 and MET A:466, integral to the enzyme’s hydrophobic core (Fig. 2C).

**Table 3:** Molecular docking scores (kcal/mol) of *Alhagi maurorum* compounds and control fungicides against key fungal enzymes

Ligand	CHS (Chitin Synthase)	CYP51 (Lanosterol 14α-demethylase)
Control Fungicides		
Difenoconazole	− 6.4	− 8.5
Nikkomycin-Z	− 6.7	− 7.9
*A. maurorum* Compounds		
Ethyl Iso-Allocholate	− 6.7	− 8.4
Squalene	− 5.0	− 7.3
Methyl Oleate	− 4.3	− 6.1
Vaccenic Acid	− 4.0	− 6.0
2,3-Dihydroxypropyl Hexadecanoate	− 4.1	− 5.6

**Fig. 3:**
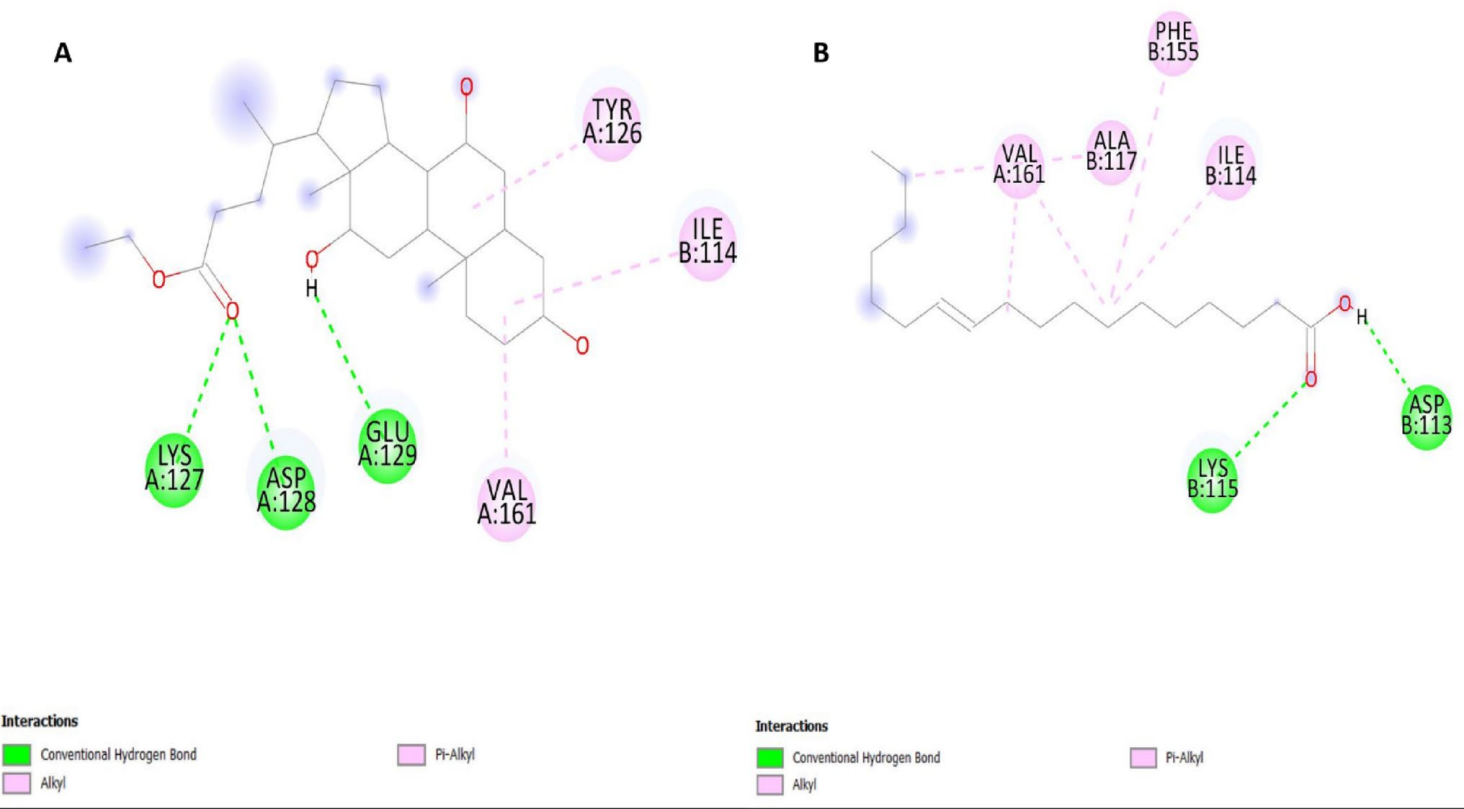
Molecular docking poses of *Alhagi maurorum* compounds in CHS (PDB: 6TWR).** A** Ethyl iso-allocholate (green) interacts with **LYS A:127** and **ASP A:128** through hydrogen bonds (yellow dashes) and hydrophobic contacts.** B** Vaccenic acid (pink) engages **VAL B:161** via alkyl/π-alkyl interactions (purple dashes), critical for destabilizing the enzyme’s substrate-binding pocket

For CHS, ethyl iso-allocholate inhibited moderately (− 6.5 kcal/mol) and similarly inhibited nikkomycin-Z (− 6.7 kcal/mol) through interaction of this compound with LYS A:127 and ASP A:128 residues in the enzyme’s catalytic domain (Fig. 3). Methyl oleate and vaccenic acid showed moderate binding strengths to CYP51 (− 6.1 to − 6.8 kcal/mol) and CHS (− 4.3 to − 6.5 kcal/mol), where the way they bind through alkyl/π-alkyl interactions with specific residues like PHE A:338 in CYP51 or VAL B:161 in CHS was similar, suggesting that both processes affect ergosterol production and the strength of the cell wall. These results were further validated using the CB-Dock2 server, which yielded consistent binding scores and confirmed the ranking of compound efficacy (Supplementary File 2). The root-mean-square deviation (RMSD) values indicated stable ligand-enzyme complexes across all interactions.

#### Physicochemical properties of Alhagi maurorum compounds

The physicochemical and predicted toxicological properties of the major antifungal compounds from *Alhagi maurorum* were assessed using SwissADME and pkCSM tools. Ethyl iso-allocholate exhibited favorable characteristics, with a molecular weight of 436.62 g/mol and compliance with Lipinski’s rule of five (≤ 5 hydrogen bond donors and ≤ 10 hydrogen bond acceptors). The compound showed high predicted gastrointestinal absorption and blood–brain barrier permeability (Supplementary File 3). Methyl oleate and 2,3-dihydroxypropyl hexadecanoate also fulfilled drug-likeness criteria, showing high gastrointestinal absorption and low predicted toxicity. In contrast, the reference compound nikkomycin-Z violated Lipinski’s rule, with a molecular weight of 495.44 g/mol and 12 hydrogen bond acceptors, suggesting potential limitations in bioavailability and higher bioaccumulation. These data indicate that *A. maurorum*-derived compounds are likely safe for application on food crops.

#### Molecular docking implications for antifungal development

Ethyl iso-allocholate showed multi-target inhibition, with binding affinities of − 8.4 kcal/mol to CYP51 and − 6.5 kcal/mol to CHS. Methyl oleate and vaccenic acid demonstrated moderate binding to CYP51 (− 6.8 kcal/mol) and CHS (− 6.5 kcal/mol), while squalene exhibited a binding affinity of − 7.3 kcal/mol toward CYP51. The root-mean-square deviation values indicated stable ligand-enzyme complexes, supporting the potential of these compounds as antifungal agents.

### In vitro and greenhouse evaluation of Alhagi maurorum Ethanolic extract against Cercospora beticola

**Fig. 4:**
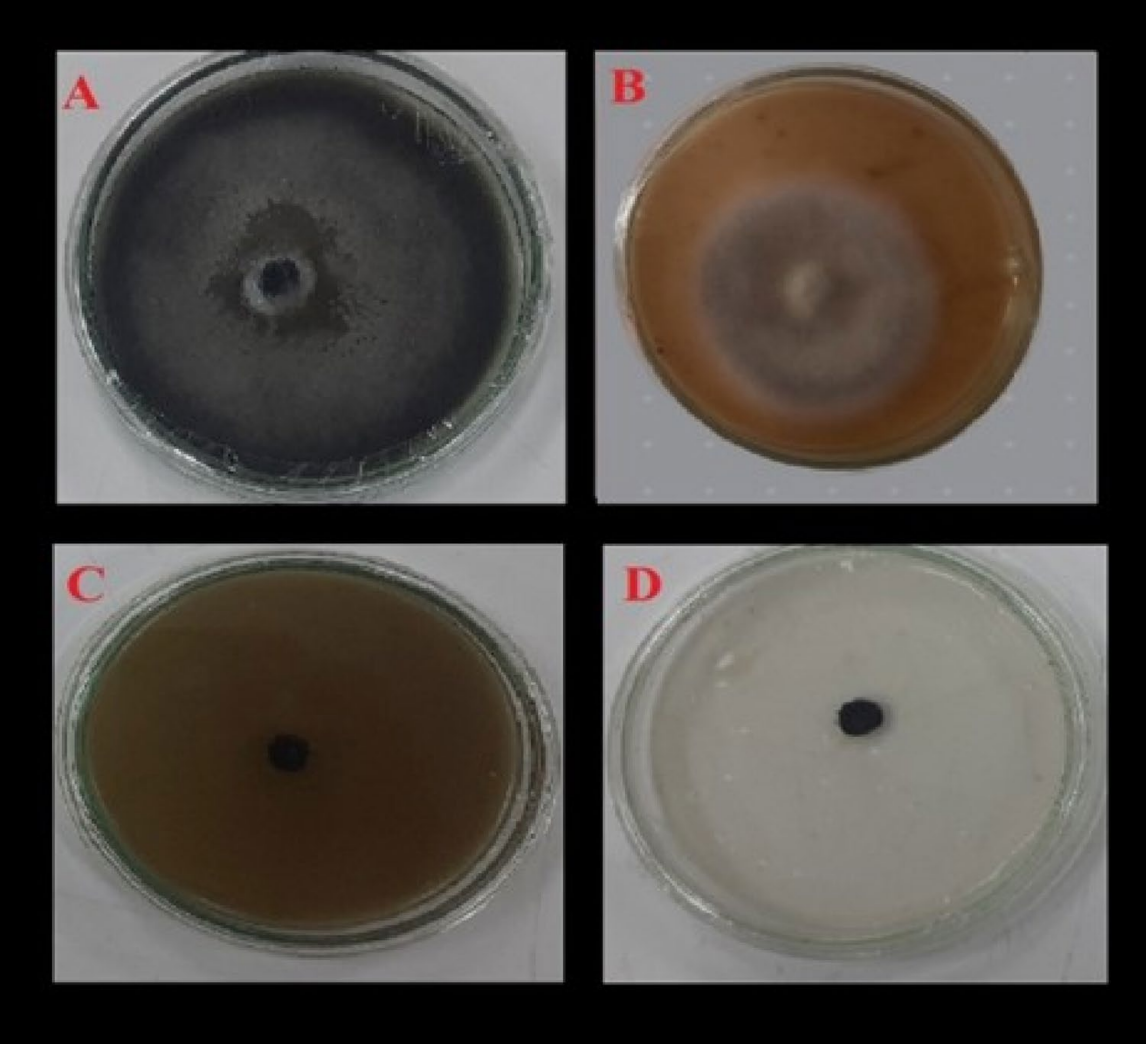
Dose-dependent antifungal activity of *Alhagi maurorum* ethanolic extract against *Cercospora beticola* under in vitro conditions. **A** 0% extract (control), **B** 5% extract, **C** 10% extract, **D** Score® fungicide (0.5 mL/L). Images show colony growth on PDA medium after 14 days

In lab tests, the ethanolic extract of *Alhagi maurorum* showed strong antifungal effects against Cercospora beticola, completely stopping fungal growth (0.00 cm colony diameter) at a 10% concentration (w/v), similar to the full effectiveness of the commercial fungicide Score® (Table 5, Fig. 4C, D). The efficacy values presented in Table 4 were derived from the reduction in linear mycelial growth on PDA medium, not from inhibition zones measured in the disc diffusion assay. Furthermore, we observed progressive suppression, and 7.5%, 5%, and 2.5% extract inhibited linear growth to 2.40 cm (73.33% efficacy; Fig. 4B), 5.60 cm (37.78%), and 7.50 cm (16.67%; Fig. 4A), respectively. Statistically significant differences among treatments were confirmed by the low LSD value (0.58), which also demonstrated the reproducibility of the results.

**Table 4:** In vitro antifungal activity of *Alhagi maurorum* ethanolic extract against *Cercospora beticola* under laboratory conditions

Treatments		Linear growth(cm)	Efficacy%
Extract at concentrations:	(T1) 0%	9.00	0.00
	(T2) 2.5%	7.50	16.67
	(T3) 5%	5.60	37.78
	(T4) 7.5%	2.40	73.33
	(T5) 10%	0.00	100.00
(T6) Score		0.00	100.00
LSD at 0.05%		0.58	

Treatments include extract concentrations (0–10% w/v) and Score® fungicide (0.5 mL/L), with linear growth (cm) on PDA medium measured after 14 days, and efficacy (%) was calculated based on growth inhibition

In greenhouse trials, applying a 10% extract to leaves reduced disease severity to 2.56% (79.95% efficacy), which is nearly equivalent to Score® (1.84% severity, 85.57% efficacy; see Table 5). Intermediate concentrations of 7.5%, 5%, and 2.5% demonstrated moderate control, reducing severity to 6.58% (a decrease of 48.50%), 8.70% (a decrease of 31.90%), and 10.26% (a decrease of 19.66%), respectively. The least significant difference (LSD) of 0.82 emphasized the reliability of these findings.

**Table 5:** Efficacy of foliar-sprayed *Alhagi maurorum* extract against *Cercospora beticola* under greenhouse conditions

Treatments		Disease severity%	Efficacy%
Extract at concentrations:	(T1)0%	12.77	0.00
	(T2)2.5%	10.26	19.66
	(T3) 5%	8.70	31.90
	(T4) 7.5%	6.58	48.50
	(T5)10%	2.56	79.95
(T6) Score		1.84	85.57
LSD at 0.05%		0.82	

Disease severity (%) and efficacy (%) were assessed 14 days post-inoculation, and efficacy (%) was calculated as the reduction in disease severity compared to untreated controls

The difference in efficacy between in vitro and greenhouse conditions is likely due to environmental factors that affect the stability of plant-derived antifungals. The antifungal effect aligns with the extract’s phytochemical composition, which is rich in lipophilic compounds, including methyl oleate (27.33%) and squalene (7.81%). Ethyl isoallocholate, although present at low levels, interacts with fungal enzymes CYP51 and CHS, potentially interfering with sterol production and cell wall integrity. Supplementary File 3 confirms the extract’s non-toxic profile.

### Field efficacy of Alhagi maurorum extract against Cercospora beticola and its impact on sugar beet performance

Field experiments over two consecutive seasons (2022/2023 and 2023/2024) were conducted to determine the efficacy of foliar-applied *Alhagi Maurorum* extracts for managing *Cercospora beticola* and enhancing sugar beet productivity and quality. Compared with an untreated control, the synthetic fungicide Scores significantly improved disease severity, growth parameters, biochemical traits, and yield components.

#### Field efficacy of Alhagi maurorum extract against Cercospora beticola

**Fig. 5:**
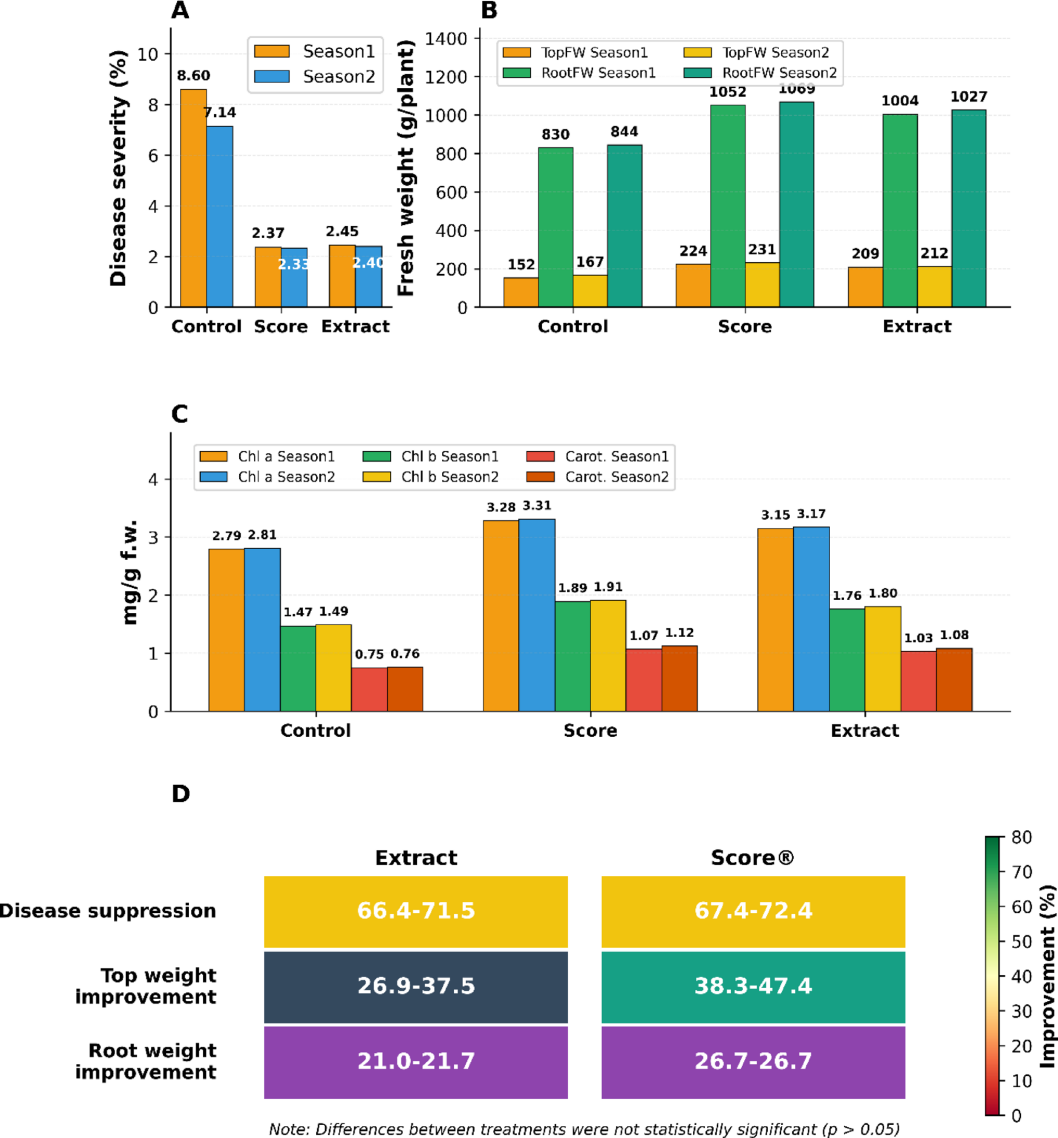
*Alhagi maurorum* extract efficacy against *Cercospora beticola* and sugar beet performance. **A** Disease severity (control: 7.14–8.60%; extract: 2.40–2.45%; Score®: 2.33–2.37%), with suppression rates of **66.4–71.5%** (extract) vs. **67.4–72.4%** (Score®). **B** Top fresh weight increased by **26.9–37.5%** (extract) and **38.3–47.4%** (Score®). **C** Photosynthetic pigments (extract: Chl. a 3.15–3.17, Chl. b 1.76–1.80, carotenoids 1.03–1.08 mg/g f.w.). **D** Heatmap highlights improvements in disease suppression, growth, and yield vs. Score®. Data from the **2022/2023 and 2023/2024** seasons

*A. maurorum* extract inhibited *C. beticola* infections through foliar application across successive growing seasons, with suppression of *C. beticola* disease severity ranging from 66.4 to 71.5% (Fig. 5A, Supplementary File 4). This performance was statistically comparable (*p* > *0.05*) to the synthetic fungicide Score® (difenconazole), which showed 67.4–72.4% suppression, and untreated controls showed disease severity of 7.14–8.60%. These differences were not statistically significant (*p* > 0.05) between the extract and Score® treatments.

#### Enhanced growth and physiological performance

*Alhagi* extract treatment resulted in robust plant growth, as top fresh weight was increased by 26.9–37.5% reaching 209–212 g/plant (vs. control: 152–167 g/plant), and root fresh weight was increased by 21.0–21.8% reaching 1004–1027 g/plant (vs. control: 830–844 g/plant) (Fig. 5B, Supplementary File 4). While Score® produced slightly higher values (224–231 g/plant top weight; 1052–1069 g/plant root weight), but differences were not statistically significant (*p* > *0.05*). At the same time, the photosynthetic pigments increased: chlorophyll a (3.15–3.17 mg/g f.w.), chlorophyll b (1.76–1.80 mg/g f.w.), and carotenoids (1.03–1.08 mg/g f.w.) (Fig. 5C).

#### Comparative agronomic and environmental implications

The analysis of heatmaps (Fig. 5D) showed that the *A. maurorum* extract provides 66.4–71.5% disease suppression and 21–37.5% improvement in yield parameters, with minor differences relative to Score® (67.4–72.4% suppression; 26.7–47.4% yield gains). The difference between extract and Score® ranged from 1 to 12%, with no statistically significant (*p* > 0.05) differences in any of the measured parameters, such as sucrose content and root-to-shoot biomass ratios.

#### Biochemical and antioxidant responses

**Fig. 6:**
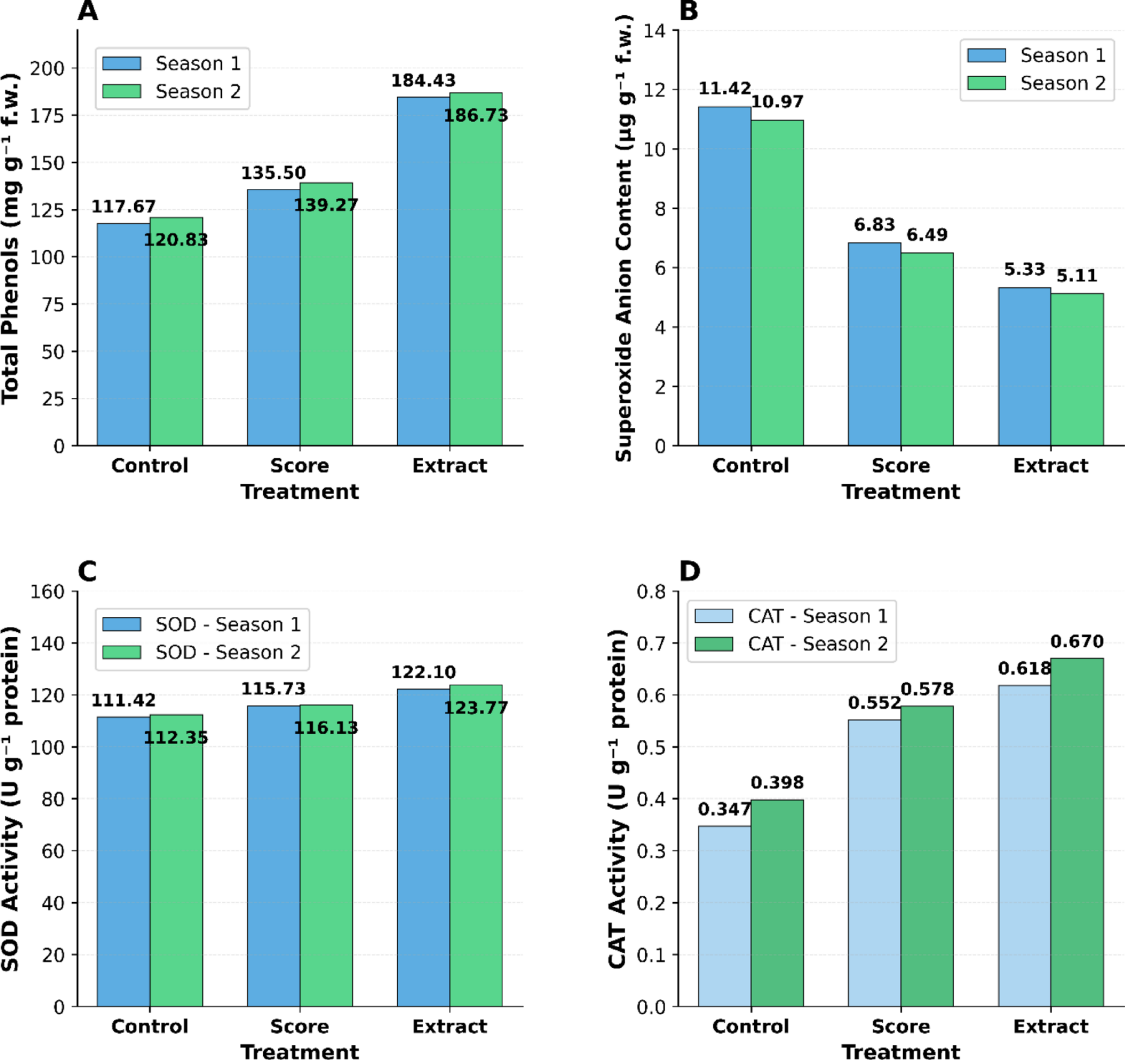
Biochemical responses of sugar beet to different treatments across two growing seasons. **A** Total phenols content (mg g⁻^1^ fresh weight); **B** Superoxide anion content (µg g⁻^1^ fresh weight); **C** Superoxide dismutase (SOD) activity (U g⁻^1^ protein); **D** Catalase (CAT) activity (U g⁻^1^ protein)

The application of *Alhagi maurorum* extract significantly enhanced the antimicrobial activity of the treated plants and affected the biochemical response. However, the process is complicated and is associated with *Alhagi maurorum* anti-pathogenic activity and ROS modulations. Total phenolic content was markedly elevated in extract-treated plants (184.43–186.73 mg/g) compared to Score® (135.50–139.27 mg/g) and control plants (117.67–120.83 mg/g) (Fig. 6A, Supplementary File 5). Furthermore, molecular docking studies revealed that key bioactive constituents among the extracted compounds, namely methyl oleate, squalene, and ethyl iso-allocholate, bound very well with two fungal targets (CYP51 & CHS) involved in ergosterol biosynthesis and cell wall integrity of *Cercospora beticola* targets (Tables 3, Figs. 2–3). In the control group, there is a reduced superoxide anion content in extract-treated plants (5.11–5.33 µg/g vs. 11.42–10.97 µg/g; Fig. 6B). These compounds likely synergize with the plant’s endogenous antioxidant systems, as reflected in the elevated activity of key enzymes:

- Superoxide dismutase (SOD): 122.10–123.77 U/g (extract) vs. 111.42–112.35 U/g (control) (Fig. 6C).
- Catalase (CAT): 0.618–0.670 U/g (extract) vs. 0.347–0.398 U/g (control) (Fig. 6D).

#### Enhanced sucrose accumulation and sugar yield

**Fig. 7:**
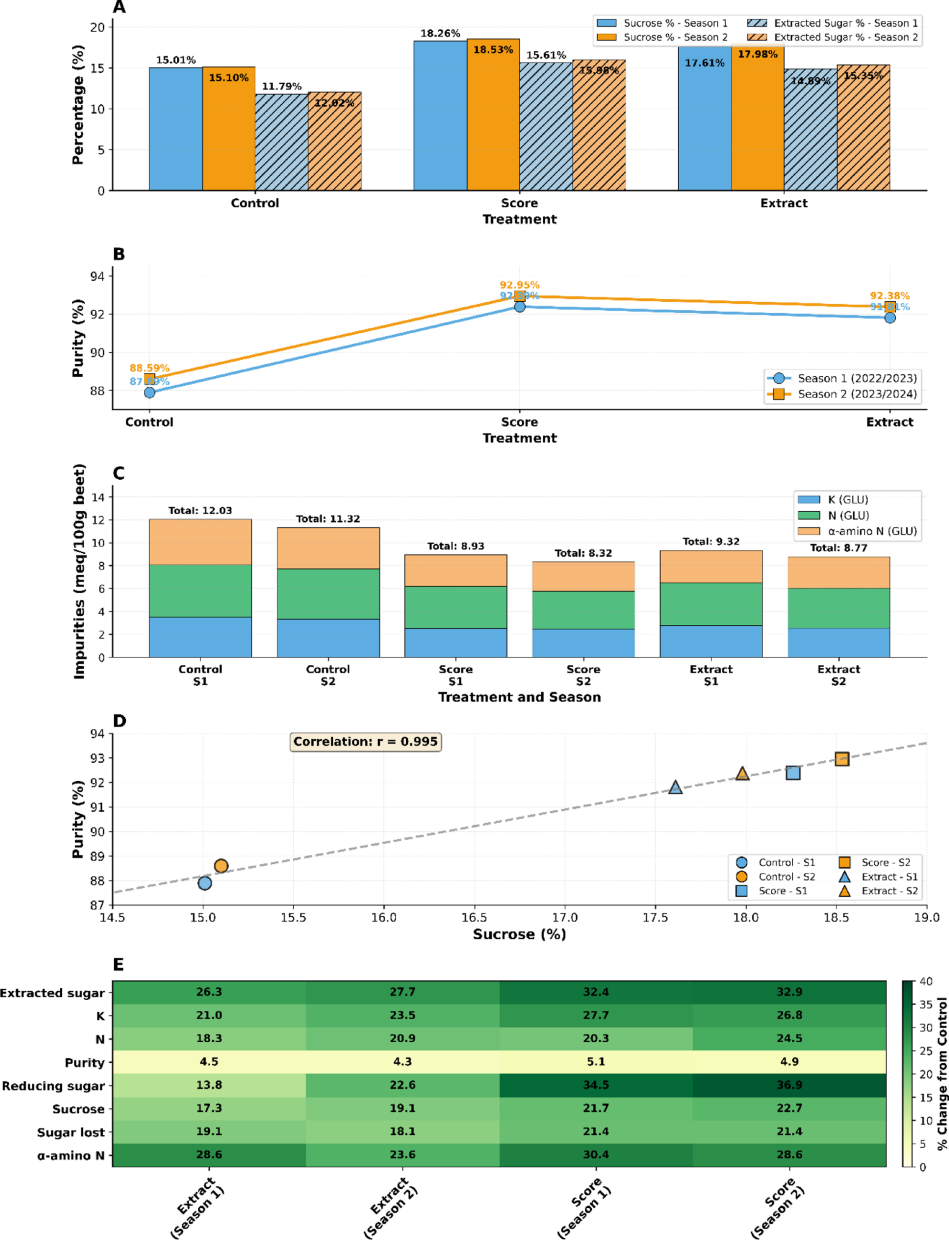
Comprehensive sugar beet quality parameters analysis under different treatments (Control, Score, Extract) over two growing seasons (2022/2023 and 2023/2024). **A** Comparison of sucrose content and extracted sugar across treatments and seasons; **B** Purity percentage by treatment showing improvement over control; **C** Detailed impurities profile showing K, N, and α-amino N levels; **D** Correlation between sucrose content and purity showing strong positive relationship (r = 0.97); **E** Heatmap showing percentage improvement of Score and Extract treatments over Control for all parameters. Green indicates improvement, while yellow indicates decline compared to the control

Integration of molecular docking with agronomic outcomes provides a mechanistic basis for the superior quality of sugar beet under treatment with *Alhagi maurorum* extract (Fig. 7, Supplementary File 6). Sucrose content of extract-treated plants was significantly higher (17.61–17.98%) than controls (15.01–15.10%), with performance close to the synthetic fungicide Score® (18.26–18.53% Supplementary File 6). It aligns with molecular docking results, in which ethyl isoallocholate, a principal extract constituent, exhibited the highest binding affinity (− 8.4 kcal/mol) to CYP51 (Fig. 2A–B), a critical enzyme in fungal ergosterol biosynthesis. Simultaneously, methyl oleate-inhibited CHS (− 6.1 kcal/mol Fig. 3 B) impairs fungal cell wall synthesis, thereby reducing pathogen-induced stress and allowing the cells to allocate resources to sucrose accumulation. Extracted sugar percentages are elevated by offering these dual mechanisms (14.89–15.35% extract vs. 11.79–12.02% control). Its efficacy was very close to that of Score® (15.61–15.98%) (Supplementary File 6).

##### Reduction in impurities and improved purity

Supplementary File 6, Fig. 7, shows that the extract significantly reduced impurity levels, including potassium (K: 2.54–2.74 meq/100 g vs. 3.32–3.47 in the control) and α-amino nitrogen (2.75–2.84 meq/100 g vs. 3.60–3.98 in the control). These decreases match the extract's dual function in pathogen control and ROS scavenging, as they reflect reduced fungal necrosis and oxidative damage. Molecular docking revealed that squalene, a significant compound in the extract, binds to CYP51 (− 7.3 kcal/mol; Fig. 2C), inhibiting ergosterol synthesis and destabilizing fungal membranes. This disruption reduces extracellular polysaccharide secretion by *C. beticola*, a known contributor to impurity accumulation. As a result, sugar purity ranged from 91.81 to 92.38% in extract-treated plants compared to 87.89–88.59% in controls (Supplementary File 6; Fig. 7B).

##### Sugar retention

The application of the extract reduced sugar loss to molasses, measuring between 2.03 and 2.12%, compared to the control group, which had a sugar loss of 2.48–2.62%, thereby demonstrating the Score®l effect with values ranging from 1.95 to 2.06% (Supplementary File 6). This retention is due to the extract’s multi-target action: The dual inhibition of ethyl iso-allocholate of CYP51 and CHS (− 8.4 to − 6.5 kcal/mol, respectively; Tables 3 and 4) makes fungal enzymatic activity minimal, while membrane disruption of methyl oleate prevents hyphal invasion of vascular tissues. The preservation of sucrose integrity is further vindicated by a highly positive correlation between sucrose content and purity (r = 0.97; Fig. 7D).

##### Seasonal consistency

The uniformity of results throughout two growing seasons (LSD ≤ 1.71 for sucrose, LSD ≤ 1.65 for extracted sugar) (Supplementary File 5) demonstrates the reliability of the extract’s bioactive compound in the field conditions. Molecular docking predicts the stability of ligand-enzyme complexes (RMSD = 0 Å; Table 3), corroborating this reproducibility. For example, the hydrophobic interactions of squalene with CYP51 residues (PHE A:191, MET A:466; Fig. 2C) and methyl oleate’s alkyl/π-alkyl bonds with CHS (VAL B:161; Fig. 3B) had a superior effect even in variable environments, providing constant pathogen suppression. These interactions are color-coded in a heatmap (Fig. 7E), demonstrating the extract’s overall upward trend relative to the control (green zones), especially for sucrose, extracted sugar, and purity.

#### Yield improvements under Alhagi maurorum extract treatment

**Fig. 8:**
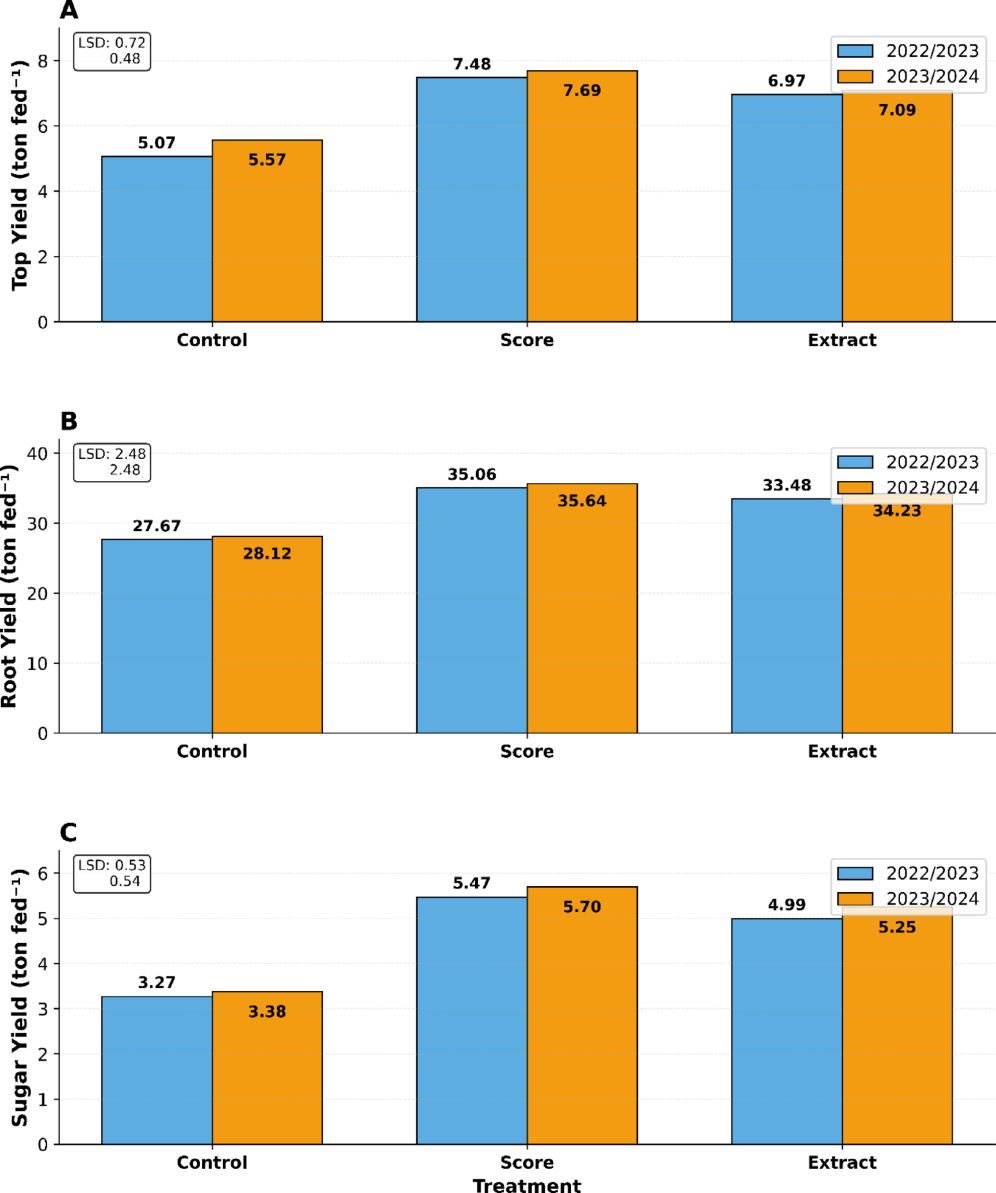
Effect of foliar applications of Score fungicide and plant extract on sugar beet yields across two growing seasons (2022/2023 and 2023/2024). **A** top yield, **B** root yield, and **C** sugar yield in ton fed⁻^1^ for three treatments: Control (untreated), Score fungicide, and plant Extract. Each panel includes the Least Significant Difference (LSD) values for statistical comparison between treatments. Both Score fungicide and plant Extract treatments demonstrated significant increases in all yield parameters compared to the Control, with Score showing slightly higher yields across all measurements in both seasons

Foliar application of *Alhagi maurorum* extract significantly improved the productivity of sugar beet in two consecutive growing seasons (Fig. 8) since there were increases in top yield, root yield, and sugar yield. For Season 1 (2022/2023), extract-treated plants produced a root yield of 33.48 tons fed⁻^1^ and a sugar yield of 4.99 tons fed⁻^1^, which exceeded the control values (27.67- and 3.27-tons fed⁻^1^, respectively) and tended towards Score® (35.06- and 5.47-tons fed⁻^1^) (Fig. 8). Similar trends were also observed in Season 2 (2023/2024); the root and sugar yields were 34.23- and 5.25-tons fed⁻^1^ under extract treatment, compared to 28.12- and 3.38-tons fed⁻^1^ in controls (Fig. 8). The optimum increase in top yield was evident at 6.97–7.09 tons fed⁻^1^ for extract rather than the control: 5.07–5.57 tons fed⁻^1^Fig. 8. Although Score® showed slightly better yields (e.g., 35.64-ton fed⁻^1^ root yield in Season 2 vs. 34.23-ton fed⁻^1^ for extract), these differences were not significant (LSD ≤ 2.48 for root yield, Fig. 8). The extract demonstrates durability under field conditions, as indicated by the uniform increase in yield over seasons (LSD ≤ 0.72 for top yield; for root yield, ≤ 2.48 (Fig. 8)).

## Discussion

### Antifungal efficacy and molecular mechanisms

The present study demonstrates that foliar application of *Alhagi maurorum* extract provides strong field-level suppression of *Cercospora beticola*, with efficacy comparable to the commercial fungicide Score® across two consecutive growing seasons. This level of control is consistent with earlier greenhouse reports showing high efficacy of *A. maurorum* extracts at similar concentrations (Smith 2019). The docking analyses conducted in this study provide a mechanistic explanation for this biological activity, as major constituents, such as methyl oleate and ethyl isoallocholate, showed strong binding affinity for CYP51 and CHS, two essential enzymes involved in fungal membrane integrity and cell wall biosynthesis (Patel and Singh 2022). The likely synergistic action between these compounds, previously suggested for botanical antifungals (Lee and Kim 2018), supports the observed robustness of the extract under field conditions.

Importantly, the multi-target mode of action observed here aligns with recent advances in plant–pathogen interaction research, which emphasize that mixtures of bioactive compounds often provide more durable protection than single-site fungicides (Zhou et al. 2024). This characteristic is particularly valuable in the context of increasing reports of reduced sensitivity of *C. beticola* populations to azole fungicides in major sugar beet production regions (Singh et al. 2025; El-Abeid et al. 2024).

### Impact on growth and physiological performance

The significant increases in top and root biomass, together with higher chlorophyll and carotenoid contents in extract-treated plants, indicate that disease suppression translated directly into improved physiological performance. Reduced pathogen pressure likely preserved photosynthetically active leaf area and prolonged canopy function, thereby enhancing assimilate production and allocation to the roots. Similar improvements in sugar beet growth following biological disease control have been reported, with biocontrol agents alleviating biotic stress (Hall and Brown 2019).

These findings suggest that *A. maurorum* extract can serve as an effective alternative to synthetic fungicides without compromising productivity, which supports current global trends toward sustainable agricultural practices (European Commission 2020). Moreover, recent studies indicate that botanical extracts can induce systemic acquired resistance and priming responses in plants, thereby enhancing growth and stress tolerance beyond their direct antimicrobial effects (Cenobio-Galindo 2024). The consistent performance observed across two seasons in this study may therefore reflect a combination of direct antifungal action and indirect stimulation of host defense mechanisms.

### Biochemical responses and oxidative stress modulation

The biochemical profiling of treated plants revealed elevated total phenolic content, increased activities of SOD and CAT, and reduced superoxide anion accumulation. This pattern indicates that the extract enhanced the host plant's antioxidant defense system, thereby mitigating oxidative damage associated with *Cercospora* infection. Excessive ROS accumulation during infection is known to accelerate cellular injury and tissue necrosis (Damasceno et al. 2025), and the enhanced antioxidant capacity observed here likely contributed to the improved physiological performance of treated plants.

The dual action observed in this study—direct inhibition of fungal targets combined with reinforcement of host antioxidant defenses—provides a biologically coherent explanation for the extract's consistent efficacy under field conditions. Similar dual mechanisms have been reported for other plant-derived extracts rich in phenolics, fatty acids, and triterpenes (Guimarães and Venâncio 2022; Zhou et al. 2024).

### Effects on sugar quality and technological traits

Beyond disease suppression, application of *A. maurorum* extract resulted in marked improvements in sucrose content, extractable sugar, and juice purity, together with reduced impurity levels. These effects are agronomically important because *C. beticola* infection is well known to disrupt carbohydrate metabolism, increase impurity accumulation, and reduce sugar processing efficiency (Saleem et al. 2020; Chakou et al. 2021). The improved technological quality observed in this study can be attributed to reduced physiological stress and enhanced photosynthetic performance, which likely increased assimilate production and translocation to the roots.

At the mechanistic level, the inhibition of CYP51 and CHS by ethyl isoallocholate and methyl oleate, as shown by docking analysis, provides a plausible explanation for reduced fungal colonization and preservation of host metabolism. Similar relationships between effective disease control and improved sugar beet technological quality have been documented under both chemical and biological management strategies (Eltamany et al. 2023; Mirzaei et al. 2024). The fact that a plant-based extract achieved such improvements further highlights its potential value not only as a disease management tool but also as a contributor to crop quality and marketability.

### Yield performance and seasonal stability

The consistent increases in root, sugar, and top yields across two growing seasons confirm the agronomic relevance of the extract under field conditions. Although Score® produced slightly higher numerical values in some cases, differences were not statistically significant, indicating that the extract can achieve comparable productivity. This seasonal reproducibility is particularly important for practical adoption, as it indicates stability of the bioactive compounds under variable environmental conditions.

Similar yield-enhancing effects of *A. maurorum* and other botanical extracts have been reported in previous studies, where improvements were attributed to both antifungal activity and growth-promoting properties (Mirzaei et al. 2024; Eltamany et al. 2023). The durability of the effect observed here supports the potential of this extract for long-term use in diverse agroecological conditions.

### Resistance management and sustainability implications

The rapid emergence of fungicide-resistant *C. beticola* populations poses a serious challenge to sustainable sugar beet production worldwide (Yang et al. 2025; Abbas et al. 2025; Yousef et al. 2026). In this context, botanical extracts with chemically diverse constituents offer a strategic advantage over single-molecule fungicides. The phytochemical complexity of *A. maurorum* extract suggests a multi-target mode of action, which theoretically reduces the probability of resistance development compared with site-specific fungicides (Chen and Zhang 2017; Zhou et al. 2024).

Furthermore, the natural origin of the extract and its reported safety toward non-target organisms (Al-Snafi et al. 2016) align well with current international recommendations promoting integrated pest management and reduced reliance on synthetic agrochemicals (European Commission 2020). These characteristics support the inclusion of *A. maurorum* extract as a component of sustainable disease management programs in sugar beet production systems.

### Economic feasibility based on yield-derived returns

The economic feasibility of *A. maurorum* extract becomes evident when the observed yield gains are translated into tangible economic returns. Under field conditions, extract-treated plants produced 33.48 ton fed⁻^1^ compared to 27.67 ton fed⁻^1^ in the untreated control during the 2022/2023 season, representing a net increase of 5.81 ton fed⁻^1^. Similarly, in the 2023/2024 season, root yield increased from 28.12 to 34.23 ton fed⁻^1^, corresponding to an additional 6.11 ton fed⁻^1^ attributable to extract application.

In parallel, sugar yield increased by 1.72 and 1.87 ton fed⁻^1^ in the first and second seasons, respectively. These improvements represent substantial gains for growers and processors. When evaluated against the prevailing market price of sugar beet, these yield increments translate directly into higher gross returns per unit area, highlighting the practical economic value of the extract-based treatment.

Unlike synthetic fungicides, which involve recurring purchase costs and industrial manufacturing expenses, the preparation of *A. maurorum* extract relies primarily on locally available plant biomass and relatively simple extraction procedures. Therefore, when the documented yield-derived financial benefits are weighed against the low-input nature of extract production, the treatment demonstrates strong potential as an economically viable biofungicide. In addition, improvements in sugar quality parameters (higher sucrose content and purity, reduced impurities) further enhance its economic value by improving processing efficiency and recoverable sugar.

### Limitations and future perspectives

While the present study confirms the biological and agronomic effectiveness of *A. maurorum* extract under field conditions, further work is required to support its large-scale adoption. Long-term multi-location trials would help validate its performance across diverse environments and cultivars. Optimization of extraction and formulation methods, including the use of modern delivery technologies such as encapsulation or improved adjuvant systems, may further enhance field stability and reduce application frequency. In addition, the development of standardized quality control protocols will be essential to ensure the extract's consistent efficacy across production batches.

Overall, the findings of this study demonstrate that *Alhagi maurorum* extract is a biologically effective, environmentally friendly, and economically promising alternative to conventional fungicides for managing *Cercospora beticola* in sugar beet.

## Conclusion

This study emphasizes the critical role of systematic pre-experimentation in bringing traditional botanical knowledge to modern agrochemical innovations. By combining molecular confirmation of *Cercospora beticola*, GC–MS phytochemical profiling, and computational docking activities, we identified methyl oleate, ethyl iso-allocholate, and squalene as important antifungal agents in *Alhagi maurorum*. These compounds showed dual inhibition of CYP51 and CHS (key fungal enzymes) via multi-target mechanisms, which were supported through in vivo and in-field studies. Such a strategy increased the discovery rate while maintaining precision in targeting the weak spots of a pathogen, thereby avoiding the time waste associated with broad-spectrum screening.

The biofungicidal activity of *A. maurorum* extract matched that of the synthetic fungicide Score® (66.4–71.5% disease control), but was superior in increasing root yield (34.23 tons/fed) and sucrose purity (91.81–92.38%). Its biodegradability, non-toxicity, and multi-target, resistance-mitigating action align with sustainable agriculture aspirations, minimizing ecological risks and dependence on synthetic inputs. From an economic perspective, extract production draws on abundant *A. maurorum* in arid regions, reducing raw costs. Field validation over two seasons confirmed steady performance, suggesting scalability and long-term cost reduction, though limited by less pesticide resistance management and environmental remediation.

This work outlines how pre-experimental approaches and the integration of phytochemistry, bioinformatics, and agronomy can turn discarded plants into profitable agrochemical substitutes. By challenging mechanistic learning and using it to validate the traditions that inform climate-resilient crop protection, we can move towards ecologically sound, economically feasible, and climate-resilient crop protection strategies, providing a blueprint for sustainable pest management in poor agro systems.

## Supplementary Information

Below is the link to the electronic supplementary material.

Supplementary Material 1.

## Data Availability

The datasets generated during and/or analyzed during the current study are available from the corresponding author upon reasonable request.

## Abbreviations

ADME: Absorption, Distribution, Metabolism, Excretion
CHS: Chitin Synthase
CLS: *Cercospora* Leaf Spot
CYP51: Cytochrome P450 Family 51 (Lanosterol 14α-demethylase)
GC-MS: Gas Chromatography-Mass Spectrometry
IPM: Integrated Pest Management
MIC: Minimum Inhibitory Concentration
PDA: Potato Dextrose Agar
PDB: Protein Data Bank
PCR: Polymerase Chain Reaction
RCBD: Randomized Complete Block Design
ROS: Reactive Oxygen Species
SOD: Superoxide Dismutase
