# Supplementary material for: Molecular docking-guided discovery of Alhagi maurorum metabolites as dual-target biofungicides against Cercospora leaf spot in sugar beet
Source: Physiol Mol Biol Plants. 2026 Mar 24;32(4):803–22. doi: 10.1007/s12298-026-01728-0 (PMC13125486; doi:10.1007/s12298-026-01728-0)
Supplement: Supplementary file 1 — Supplementary Material 1. [file 12298_2026_1728_MOESM1_ESM.docx]

**Supplementary File 1**:  Molecular interactions between ligands and key residues of CYP51 and CHS enzymes, including bond types (conventional, pi-pi, alkyl/pi-alkyl) and critical amino acids involved in binding.

| Enzyme | Ligand | Bond | Amino Acid |
| --- | --- | --- | --- |
| CYP51 | Difenoconazole (control) | Conventional | TYR A:22 |
|  |  | Pi-Pi T-shaped | PHE A:338, PRO A:188 |
|  |  | Alkyl/ Pi-Alkyl | ILE A:189, TYR A:76, MET A:466, LEU A:334, LEU A:79 |
|  | Nikkomycin-Z (control) | Conventional | ARG A:339, HIS A:426, TYR A:90, TYR A:76 |
|  |  | Pi-Pi T-shaped | PHE A:84 |
|  |  | Alkyl/ Pi-Alkyl | LEU A:334, MET A:466, PHE A:338 |
|  |  | Carbon-Hydrogen Bond | GLY A:261 |
|  | 2 3-Dihydroxypropyl_Hexadecanoate | Alkyl/ Pi-Alkyl | MET A:466, PRO A:188, PHE A:338, LEU A:79, LEU A:334, LEU A:45, TYR A:22 |
|  |  | Carbon-Hydrogen Bond | TYR A:76 |
|  |  | Conventional | ARG A:339, HIS A:426, TYR A:90 |
|  |  | Pi-Sigma | PHE A:191 |
|  | Ethyl_Iso-Allocholate | Conventional | CYS A:428, TYR A:90 |
|  |  | Alkyl/ Pi-Alkyl | TYR A:76, LEU A:334, ILE A:89, LYS A:101, ILE A:429, ILE A:262 |
|  | Methyl-Oleate | Alkyl/ Pi-Alkyl | PHE A:191, PRO A:188, PHE A:338, MET A:466, LEU A:79, LEU A:334, TYR A:76, TYR A:90, TYR A:22, HIS A:335, LEU A:46, LEU A:45 |
|  | Squalene | Pi-Sigma | PHE A:191 |
|  |  | Alkyl/ Pi-Alkyl | PHE A:338, MET A:466, LEU A:79, LEU A:334, TYR A:76, TYR A:90, PRO A:188, LEU A:45, LEU A:97, ILE A:89, ILE A:262 |
|  | Vaccenic-Acid | Conventional | TYR A:22 |
|  |  | Pi-Sigma | TYR A:76 |
|  |  | Alkyl/ Pi-Alkyl | LEU A:334, MET A:466, LEU A:79, PHE A:338, PRO A:188, LEU A:45, PHE A:191 |
| CHS | Difenoconazole (control) | Alkyl/ Pi-Alkyl | ALA A:117, ILE A:114, VAL B:161, LYS A:110, PHE A:155, LEU A:112 |
|  |  | Carbon-Hydrogen Bond | ASN B:166, LYS A:115 |
|  | Nikkomycin-Z (control) | Conventional | LYS B:115, ASN A:166, GLU A:167 |
|  |  | Pi-Alkyl | TYR A:126 |
|  |  | Pi-Anion | GLU A:164 |
|  | 2 3-Dihydroxypropyl_Hexadecanoate | Conventional | GLU B:167, ASN B:166 |
|  |  | Alkyl/ Pi-Alkyl | PHE A:155, TYR B:126, VAL B:161 |
|  | Ethyl_Iso-Allocholate | Conventional | LYS A:127, ASP A:128, GLU A:129 |
|  |  | Alkyl/ Pi-Alkyl | TYR A:126, ILE B:114, VAL A:161 |
|  | Methyl-Oleate | Alkyl/ Pi-Alkyl | LYS A:115, ALA A:117, TYR B:126, PHE A:155, ILE B:114 |
|  | Squalene | Alkyl/ Pi-Alkyl | ALA A:117, LEU B:125, TYR B:126, VAL B:161, LYS A:115, ILE A:114, LEU A:112, PHE A:155 |
|  | Vaccenic-Acid | Conventional | LYS B:115, ASP B:113 |
|  |  | Alkyl/ Pi-Alkyl | VAL A:161, ALA B:117, PHE B:155, ILE B:114 |

**Supplementary File 2.** Validation docking scores (kcal/mol) from CB-Dock2 for *Alhagi maurorum* compounds and control fungicides against CYP51 and CHS.

| **Ligand** | **CHS (Chitin Synthase)** | **CYP51 (Lanosterol 14α-demethylase)** |
| --- | --- | --- |
| **Control Fungicides** |  |  |
| Difenoconazole | -6.2 | -9.1 |
| Nikkomycin-Z | -6.5 | -9.5 |
| ***A. maurorum* Compounds** |  |  |
| Ethyl Iso-Allocholate | -6.5 | -8.5 |
| Squalene | -5.6 | -7.5 |
| Methyl Oleate | -4.3 | -6.8 |
| Vaccenic Acid | -4.6 | -6.5 |
| 2,3-Dihydroxypropyl Hexadecanoate | -4.4 | -6.0 |

**Supplementary File 3:** Predicted physicochemical, ADME (absorption, distribution, metabolism, excretion), and pharmacokinetic properties of prioritized compounds from *Alhagi maurorum* extract and control fungicides, assessed using SwissADME and pkCSM tools. Key parameters include molecular weight, rotatable bonds, hydrogen-bond acceptors/donors, gastrointestinal (GI) absorption, blood-brain barrier (BBB) permeability, cytochrome P450 (CYP) enzyme inhibition, Lipinski’s rule compliance, and toxicity profiles.

| Swiss ADMET | Difenoconazole | Nikkomycin Z | Methyl Oleate | Squalene | Vaccenic Acid | 2,3-Dihydroxypropyl Hexadecanoate | Ethyl Iso-Allocholate |
| --- | --- | --- | --- | --- | --- | --- | --- |
| Molecular weight (g/mol) | 406.26 | 495.44 | 296.49 | 410.72 | 282.46 | 330.50 | 436.62 |
| Num. rotatable bonds | 5 | 9 | 16 | 15 | 15 | 18 | 6 |
| Num. H-bond acceptors | 5 | 12 | 2 | 0 | 2 | 4 | 5 |
| Num. H-bond donors | 0 | 8 | 0 | 0 | 1 | 2 | 3 |
| GI absorption | High | Low | High | - | High | High | High |
| BBB permeant | Yes | No | No | - | No | Yes | No |
| CYP1A2 inhibitor | Yes | No | Yes | - | Yes | No | No |
| CYP2C19 inhibitor | Yes | No | No | - | No | No | No |
| CYP2C9 inhibitor | Yes | No | No | - | Yes | No | No |
| CYP2D6 inhibitor | No | No | No | - | No | Yes | No |
| CYP3A4 inhibitor | No | No | No | - | No | No | No |
| Lipinski | Yes | No | Yes | - | Yes | Yes | Yes |
| Toxic or non-toxic | Non-Toxic | Toxic | Non-Toxic | Toxic | Non-Toxic | Non-Toxic | Non-Toxic |

**Supplementary File 4:**Agronomic parameters of sugar beet plants treated with *Alhagi maurorum* extract or Score® fungicide (2022/2023 and 2023/2024). Includes disease severity%, growth metrics, photosynthetic pigments, and LSD values (*p* > 0.05).

| **Treatments** | | **Disease Severity%** | **Top f.w. (g/plant)** | **Root f.w. (g/plant)** | **Photosynthetic pigments (mg/g f.w.)** | | |
| --- | --- | --- | --- | --- | --- | --- | --- |
|  |  |  |  |  | **Chl. A** | **Chl. B** | **Carot.** |
| **Season1** | **Control** | 8.60 | 152 | 830 | 2.79 | 1.47 | 0.75 |
|  | **Score** | 2.37 | 224 | 1052 | 3.28 | 1.89 | 1.07 |
|  | **Extract** | 2.45 | 209 | 1004 | 3.15 | 1.76 | 1.03 |
|  | **L.S. D** | **0.30** | **22** | **65** | **0.22** | **0.17** | **0.13** |
| **Season2** | **Control** | 7.14 | 167 | 844 | 2.81 | 1.49 | 0.76 |
|  | **Score** | 2.33 | 231 | 1069 | 3.31 | 1.91 | 1.12 |
|  | **Extract** | 2.40 | 212 | 1027 | 3.17 | 1.80 | 1.08 |
|  | **L.S. D** | **0.23** | **14** | **74** | **0.24** | **0.12** | **0.16** |

**Supplementary File 5.** Total phenolic content (mg g⁻¹ fresh weight) in sugar beet plants treated with *Alhagi maurorum* extract, Score® fungicide, and untreated controls across two growing seasons. Data ranges correspond to those visualized in Fig. 6.

| **Treatments** | | **Total phenols (mg g-1 f.w.)** | **Superoxide**  **anion content**  **(µg g-1 f.w.)** | **Antioxidant enzyme activity (Ug-1 protein)** | |  |
| --- | --- | --- | --- | --- | --- | --- |
|  |  |  |  | **SOD** | **CAT** |  |
| **Season1** | **Control** | 117.67 | 11.42 | 111.42 | 0.347 |  |
|  | **Score** | 135.50 | 6.83 | 115.73 | 0.552 |  |
|  | **Extract** | 184.43 | 5.33 | 122.10 | 0.618 |  |
|  | **L.S.D** | **12.25** | **0.75** | **3.91** | **0.063** |  |
| **Season2** | **Control** | 120.83 | 10.97 | 112.35 | 0.398 |  |
|  | **Score** | 139.27 | 6.49 | 116.13 | 0.578 |  |
|  | **Extract** | 186.73 | 5.11 | 123.77 | 0.670 |  |
|  | **L.S. D** | **14.88** | **0.99** | **3.03** | **0.083** |  |

**Supplementary File 6.**Seasonal data (2022/2023 and 2023/2024) for sucrose content, impurities (potassium, nitrogen, α-amino nitrogen), sugar loss to molasses, extracted sugar percentage, purity, and reducing sugar levels in sugar beet plants treated with Alhagi maurorum extract, Score® fungicide, and untreated controls. LSD values indicate statistical significance. The data correspond to trends in Fig. 7.

| **Treatments** | | **Sucrose %** | **Impurities**  **(meq/100 g beet)** | | | **Sugar lost**  **to molasses %** | **Extracted sugar%** | **Purity%** | **Reducing sugar%** |
| --- | --- | --- | --- | --- | --- | --- | --- | --- | --- |
|  |  |  | **K** | **N** | **α-amino N** |  |  |  |  |
| **Season1** | **Control** | 15.01 | 3.47 | 4.58 | 3.98 | 2.62 | 11.79 | 87.89 | 0.87 |
|  | **Score** | 18.26 | 2.51 | 3.65 | 2.77 | 2.06 | 15.61 | 92.39 | 0.57 |
|  | **Extract** | 17.61 | 2.74 | 3.74 | 2.84 | 2.12 | 14.89 | 91.81 | 0.75 |
|  | **L.S. D** | **1.24** | **0.47** | **0.15** | **0.56** | **0.12** | **1.15** | **0.70** | **0.03** |
| **Season2** | **Control** | 15.10 | 3.32 | 4.40 | 3.60 | 2.48 | 12.02 | 88.59 | 0.84 |
|  | **Score** | 18.53 | 2.43 | 3.32 | 2.57 | 1.95 | 15.98 | 92.95 | 0.53 |
|  | **Extract** | 17.98 | 2.54 | 3.48 | 2.75 | 2.03 | 15.35 | 92.38 | 0.65 |
|  | **L.S. D** | **1.71** | **0.22** | **0.30** | **0.42** | **0.09** | **1.65** | **1.09** | **0.04** |
